# Calculation and internal validation of a new synthetic and autocorrelate index to combine the determinants of health of a population

**DOI:** 10.1186/s13690-021-00590-8

**Published:** 2021-04-29

**Authors:** Domenico Tebala, Giovanni Domenico Tebala

**Affiliations:** 1 Italian National Institute of Statistics, Catanzaro, Italy; 2 Oxford University Hospitals NHS Foundation Trust, Headley Way, Headington, Oxford, OX3 9DU UK

**Keywords:** Health, Composite index, Autocorrelation, Determinants

## Abstract

**Background:**

The status of health of an individual and - more broadly - of a community or population is defined by the status of their determinants of health. A “systemic” approach to define the health determinants is necessary in order to explore the complex relations existing among them. This study is aimed at identifying a ‘composite systemic’ index of health to measure the impact of socioeconomic factors on public health at local level and to analyze possible spatial autocorrelations between neighboring regions.

**Results:**

A Composite Index of Health (CIH) was constructed on the basis of known indicators of socio-economic well-being by using the COMIC (COMposite Indices Creator) Software and was validated on the Italian population and a nationwide comparison has been performed.

Analysis of the determinants showed a significant direct correlation between health, environment, work and wealth and inverse correlation between health and social distress. The analysis of data from Italian provinces confirmed the South-North gradient of well-being.

**Conclusion:**

The CIH is a reliable and robust index to evaluate the health of a local population. Although it was validated on Italian data, the index can be easily adapted to any Country.

## Background

The status of health of an individual and - more broadly - of a community or population is defined by the status of their determinants of health. Their unequal distribution is a well-known problem of public health [1, 2]. Despite clear recommendations on how to best address these inequalities, there is still a lack of clarity on how to target political interventions. This can be due, at least in part, to the imperfect knowledge of what data should be considered as health indicators and, within the known factors, the role that socio-economic indicators play in the determination of the general status of health of a population. Several Authors have highlighted the obvious strict causal relation existing between socioeconomic factors and status of health, where inequalities of the first would lead to disparities in the latter [3]. The study of single determinants may not be considered completely reliable, so public health researchers are turning their attention towards a complex systems approach at local and national level, which can explain interlinked social and health inequalities [4, 5]. Such a systemic approach may allow us to study the different components of the complex health system and to get to the bottom of their intricate relationships, while obtaining the wide picture on how the system – as a whole – works [6]. This would also prompt public health professionals and politicians to plan the right interventions to tackle disparities and inequalities at local and national level.

The present study is aimed at identifying a ‘composite index of health’ (CIH) to measure the impact of socioeconomic factors on public health at provincial level through the ESWB (Equitable and Sustainable Well-Being) methodology and to analyze possible spatial autocorrelations between different regional areas.

## Methods

The CIH was calculated with the ESWB methodology considering socioeconomic factors knowingly associated with public health at local level. Furthermore, we analysed possible correlations with other provinces. In fact, data measured in a specific area (or province) can be influenced by what happens in nearby areas, generating what is commonly called “spatial autocorrelation” or “spatial interdependence”. In this regard, the LISA indicators (Local Indicator of Spatial Association) provide a local framework to the measure of autocorrelation, enabling each spatial unit (i.e., the province) to assess the degree of spatial association and similarity with the surrounding elements.

In the case of positive autocorrelation, these associations can be of the High-High type (high values observed in a territorial unit and high values also in its vicinity) or Low-Low type (low values observed in a territorial unit and low values also in its neighboring areas). Conversely, in the case of negative self-correlation, the associations will be of the High-Low or Low-High type. In all other cases, there will be no autocorrelation or non-significant autocorrelation.

For simplicity, the CIH has been constructed on the basis of the Italian provinces but should be easily adapted to any other Country.

The method of construction of the index followed these steps:

1. analysis of the theoretical framework, methodology used and indicators;
2. choice of the statistical methodology
3. statistical analysis: in order to assess the robustness of the identified method and, therefore, improve decision-making, we also completed an influence analysis to analyze the most significant indicators (software COMIC - COMposite Indices Creator by Italian National Institute of Statistics – ISTAT)
4. analysis of the results with the help of a georeferenced map of the CIH of Italian provinces and a Cluster Map LISA which shows the provinces with statistically significant values of the LISA index, classified in five categories: a) Not significant (white); b) High-High (red); c) Low-Low (blue); d) Low-High (light blue); e) High-Low (light red) (software GeoDa v.1.12).

To evaluate the status of health of a community, it is necessary to identify and analyze the various determinants that affect health and, when negative, the lack of health. It is a complex set of factors relative to the individual and to their role in the society: personal factors such as gender and age, personal behavior and lifestyle, social factors, living conditions, work, access to health services, financial status and environment. The approach used involves the construction of macro areas (pillars) by aggregating elementary indicators. Both pillars and elementary indicators have been considered non-replaceable. To construct the synthetic CIH, we adopted the indicators and polarity considered in the 2015 document “Fair and sustainable wellbeing in towns” [7] published by ISTAT and defined in Table 1.

**Table 1:** Indicators and polarity

Macro areas	Indicators	Polarity
Environment	Surface intended for urban gardens (square meters per 100 inhabitants)	+
	Total density of the green areas (protected natural areas and urban green areas) (percentage on municipal surface)	+
	Green historic density and urban parks of significant public interest (m2 to 100 m2 of built-up areas)	+
	Maximum number of days exceeding the limit for the protection of human health provided for PM10 in the provincial capitals	–
	Dispersions of the drinking water network (% values)	–
	Noise Control in which at least one has been detected exceeding the limits in the provincial capitals (per 100,000 inhabitants)	–
	Density of bike paths in the provincial capitals - (km per 100 km2)	+
	Availability of pedestrian areas in the provincial capitals (m2 per 100 inhabitants)	+
Social distress	Rate of homicides reported by police to the court for the province (per 100,000 inhabitants)	–
	Thefts in homes reported by the police to the court (per 100,000 inhabitants)	
	Robberies reported by the police to the court (per 100,000 inhabitants)	
	Rate per province road accidents (per 100,000 inhabitants)	
Culture	Number of public libraries (per 100,000 inhabitants)	+
	Number of museums, archaeological sites and monuments (per 100,000 inhabitants)	
	Visitors of non-state institutes of antiquities and art institute for (number per 1000 visitors)	
	Number of users of public libraries (per 100 inhabitants)	
Health	Life expectancy at birth by sex and province (average number of years)	+
	Infant mortality rate by sex and province (rates per 10,000 live births)	–
	Standardized mortality rate for transport accidents for people aged 15–34 by sex and province (rates per 10,000 residents)	–
	Standardized mortality rate from cancer for people 20–64 years old by gender and province (rates per 10,000 residents)	–
	Standardized mortality rate for dementia and nervous system diseases for people aged 65 and over by gender and province (rates per 10,000 residents)	–
Work	Employment rate of the population aged 20–64 years by province	+
Material well-being	Per capita GDP	+

Data used in this study are publicly available on the Italian Institute of Statistics website (https://dati.istat.it). Descriptive statistics on this huge amount of data goes beyond the scope of this paper and represent the results of the Census of the Italian population [8].

The matrix of data on Italian provinces was divided into four progressive steps:

- Selection of a set of basic indicators on the basis of an ad hoc evaluation model centred on the existence of quality requirements;
- Further selection aimed at balancing the set of indicators within the theoretical framework of the structure. Outcome indicators are impact indicators as the ultimate result of an action of a stakeholder’s activity or process;
- Calculation of synthetic indices (pillars), by making use of the more appropriate methodology to obtain usable analytical information on the health of Italian provinces;
- Processing of a final synthetic index as a rapid empirical reference concerning the degree of health of Italian provinces.

Missing values were attributed via the hot-deck imputation and, where not possible, were considered overlapping Italy’s average value.

The choice of the method of synthesis is based on the assumption of a formative measurement model, in which it is believed that the elementary indicators are not replaceable, which is to say, cannot compensate each other.

The exploratory analysis of input data was performed by calculating mean, average standard deviation and frequency, as well as building up a correlation matrix and performing a principal component analysis. The correlation matrix of r-values has been developed with the Pearson’s linear correlation analysis. *P*-value < 0.05 was considered to be statistically significant. Since this is a non-compensatory approach, the simple aggregation of elementary indicators was carried out using the correct arithmetic average with a penalty proportional to the “horizontal” variability.

Normalization of primary indicators took place by conversion into relative indexes compared to the variation range (min-max).

Attribution of weights to each elementary indicator has followed a subjective approach, opting for the same weight for each of them. Since, in some cases, the elementary indicators showed different polarity, it was necessary to reverse the sign of negative polarities by linear transformation.

Calculation of the CIH has been performed with the Adjusted Mazziotta-Pareto Index (AMPI), which is used for the min-max standardization of elementary indicators, and aggregated with the mathematical average penalized by the “horizontal” variability of the indicators themselves. In practice, the compensatory effect of the arithmetic mean (average effect) is corrected by adding a factor to the average (penalty coefficient) which depends on the variability of the normalized values of each unit (horizontal variability), or by the variability of the indicators compared to the values of reference used for the normalization.

The synthetic index of the *i-th* unit, which varies between 70 and 130, is obtained by applying, with negative penalty, the correct version of the penalty method for variation coefficient (AMPI +/−), where:  *AMPIi-=Mri-Sricvi*.

*Mri* e *Sri* are, respectively, the arithmetic mean and the standard deviation of the normalized values of the indicators of the *i* unit, and *cvi = Sri/Mri* is the coefficient of variation of the normalized values of the indicators of the *i* unit.

The correction factor is a direct function of the variation coefficient of the normalized values of the indicators for each unit and, having the same arithmetic mean, it is possible to penalize units that have an increased imbalance between the indicators, pushing down the index value (the lower the index value, the lower the level of health).

This method satisfies all requirements for the wellbeing synthesis:

- Spatial and temporal comparison
- Irreplaceability of elementary indicators
- Simplicity and transparency of computation
- Immediate use and interpretation of the obtained results
- Strength of the obtained results

An influence analysis was also performed to assess the robustness of the method and to verify if and with which intensity the composite index rankings change following elimination from the starting set of a primary indicator. This process allowed us also to analyze the most significant indicators.

The final CIH values for each province were finally depicted cartographically, to have an immediate idea of the gradient of CIH within the Italian territory.

The analysis was conducted with the COMIC software, that allows the calculation of synthetic indices and rankings, as well as the comparison of different synthetic methods to select the most suitable among them and write an effective report based upon results.

Approval by an Ethical Committee was not necessary as this study did not involve human participants but only widely available population data. For this reason, the principles of the Declaration of Helsinki [9] do not apply to this study.

## Results

**Fig. 1:**
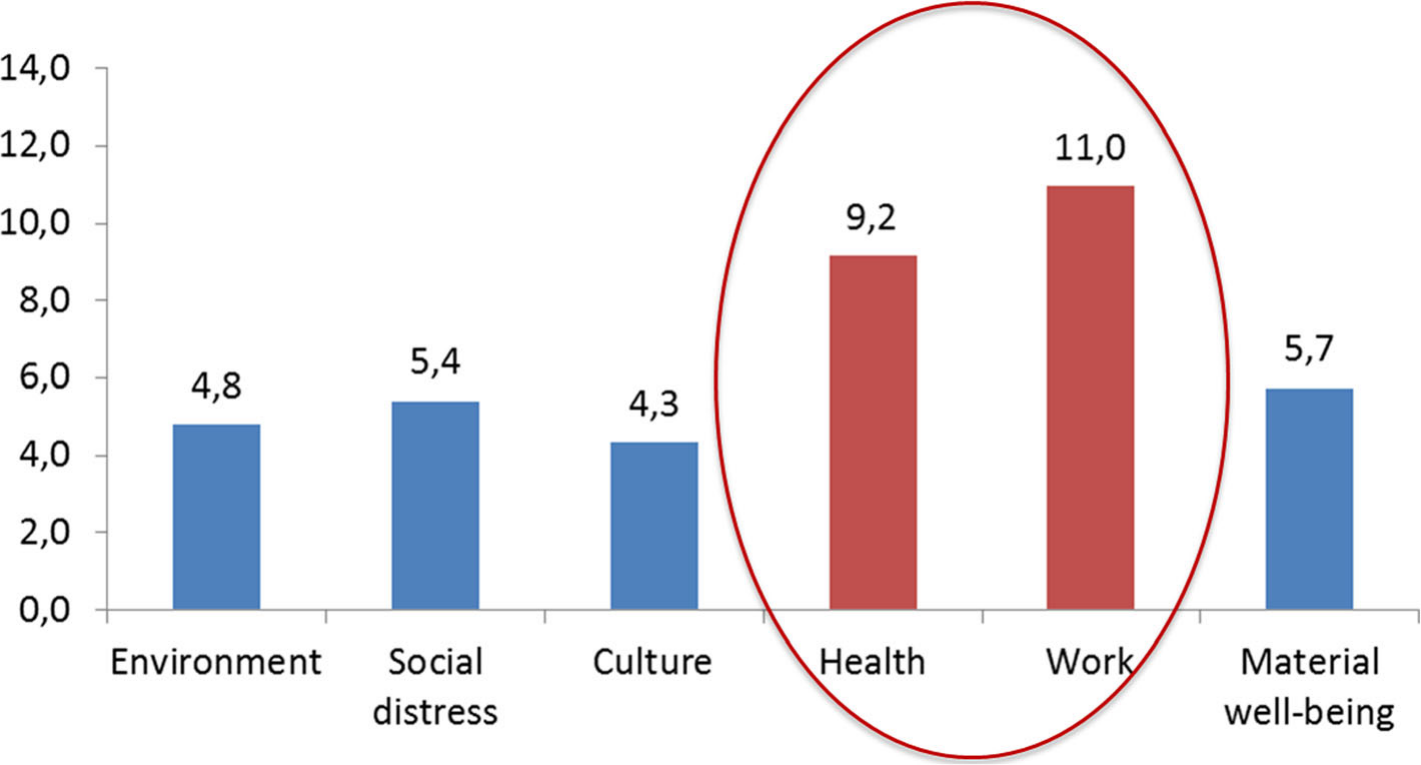
Influence Analysis: σ of the shifts for basis indicator of macro areas

The health indicators we have analysed correlate well with each other. Table 2 shows the “direct” correlations between health and environment (*r* = 0.81), work (*r* = 0.61) and material well-being (*r* = 0.57) and the “reverse” correlation between health and social distress (*r* = − 0.48). The influence analysis describes the indicators that most influence the composition of rosters in health of Italian provinces. In Fig. 1 it is evident how variables of work (σ = 10.96) and health (σ = 9.15) are the most significant.

**Table 2:** Correlation matrix of the macro areas. Pearson’s linear correlation, r-values reported, *p* < 0.05

Macro areas	Environment	Social desease	Culture	Health	Work	Material well-being
Environment	1,00					
Social desease	−0,03	1,00				
Culture	0,33	0,28	1,00			
Health	0,81	-0,48	0,32	1,00		
Work	0,50	0,01	0,53	0,61	1,00	
Material well-being	0,57	-0,08	0,09	0,54	0,24	1,00

**Fig. 2:**
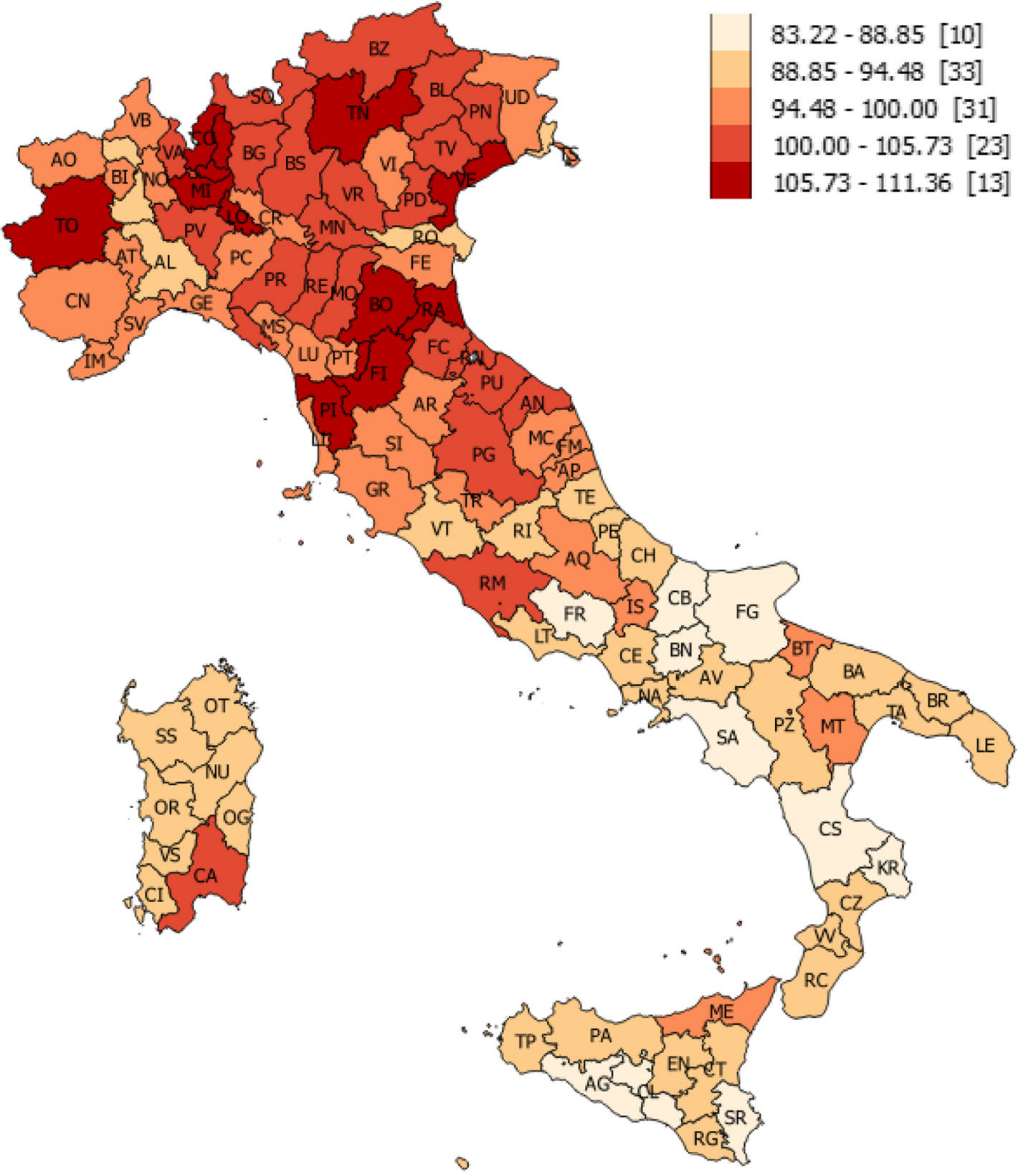
Territorial distribution of the Composite Index of Health. (In square brackets the number of provinces in that range)

The CIH distributes unequally within the Italian territory. The cartographic representation of the final CIH value, alongside with the descriptive analysis of data, yields the usual dualistic pattern South vs Centre-North of Italy as in other domains of ESWB and shows how the health of the Italian population is closely related to the socio-economic components. The best CIH performances (Fig. 2) are grouped in Tuscany (Florence, Prato and Pisa) and Lombardy (Lecco, Monza and Como), but the “healthiest” province is Trento (CIH = 111.36 - CIH for Italy = 100), thanks mainly to the cultural (123.58) and employment (117.30) indexes which in that region (Trentino) “weigh” more than the more strictly healthcare variables. Frosinone (Lazio) occupies the last position in the ranking list (CIH = 83.22), even though it is the whole South to be heavily penalized (55% of the provinces below the average belong to the South of Italy).

**Fig. 3:**
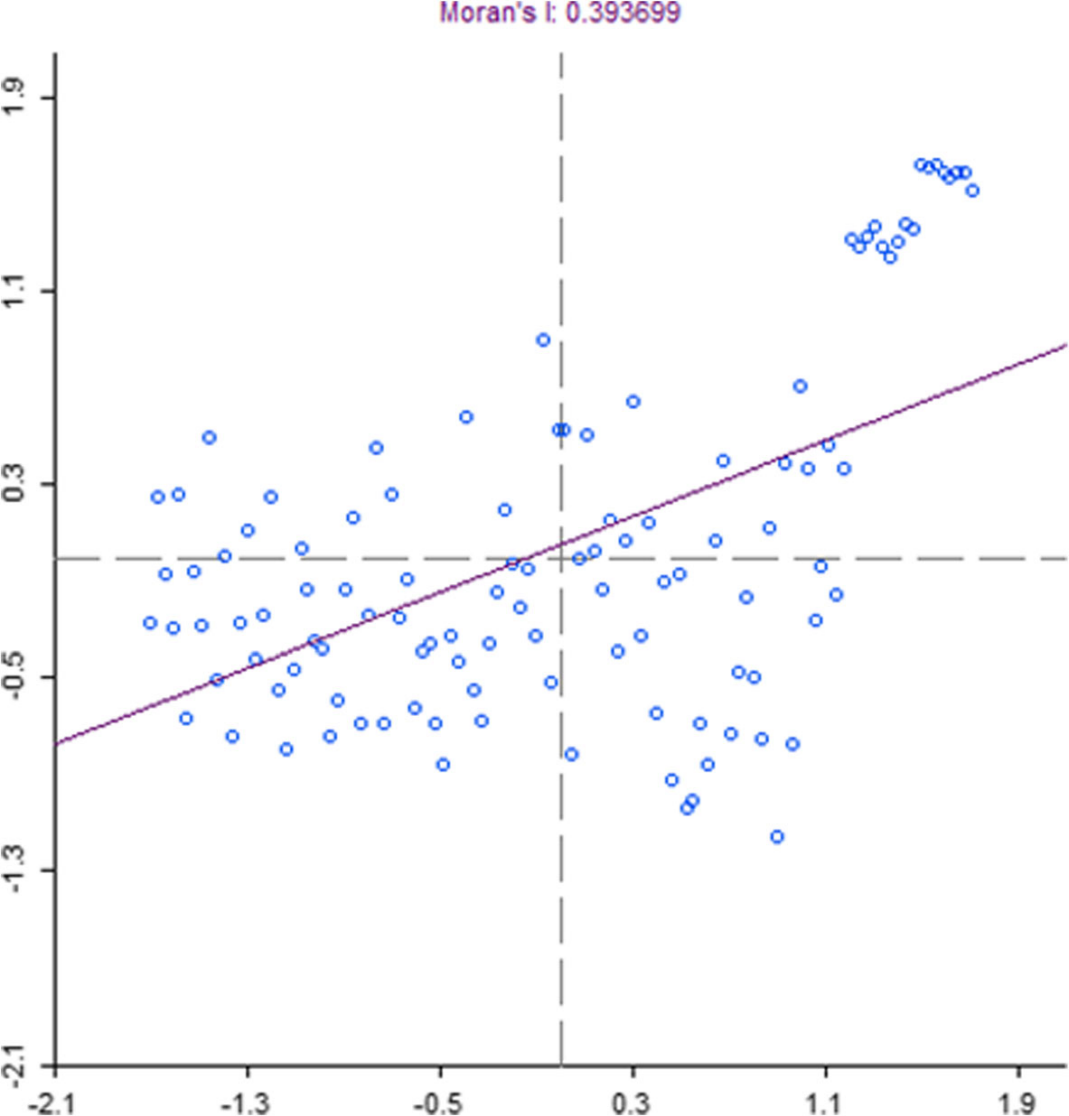
Moran’s Index

**Fig. 4:**
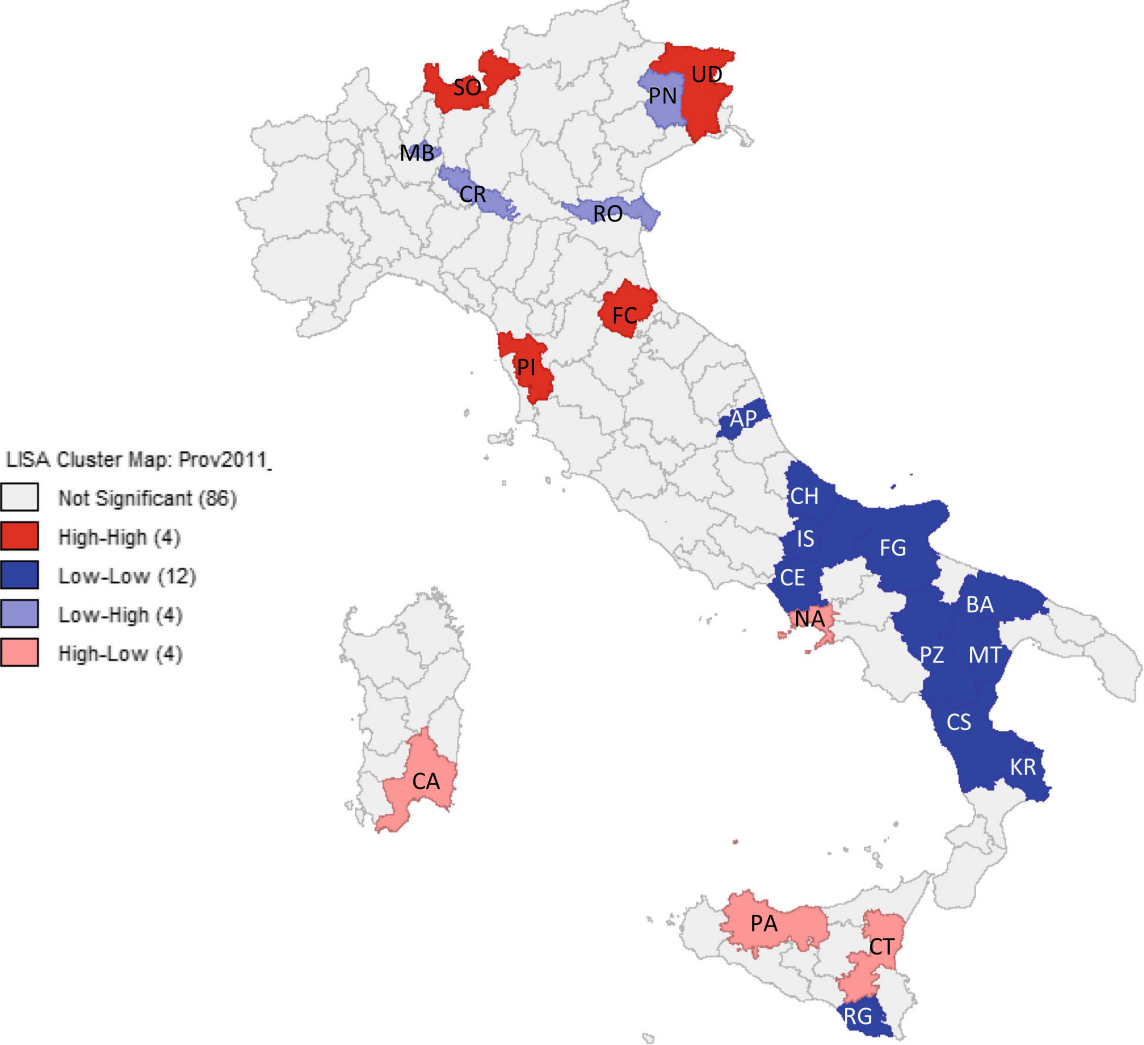
Spatial autocorrelation - LISA Cluster map Index. (in square brackets the number of provinces)

The spatial analysis shows that CIH values tend to cluster in specific areas. Our results show a good spatial interaction between neighbouring provinces (Moran’s index = 0.39) (Fig. 3). This is particularly true in the South, where there is a definite positive autocorrelation between 12 provinces, with low values observed in a province and low values in the surrounding areas (Low-Low Blue Color) (Fig. 4).

## Discussion

The study of the determinants of health is crucial to comprehend the reasons of the status of health of a population, at every level, and to plan political interventions to address inequalities and substandard performance.

The Human Development Index (HDI) has been introduced by the United Nations in 1990 to replace the gross domestic product (GDP) as unidimensional measure of the wealth of a Country [10]. The HDI measures the human development in a specific area by considering life expectancy, expected years of schooling and pro-capita gross income at national level. The cartographic representation of the HDI shows an expected gradient, where “developed” Countries score higher than “developing’ Countries [11]. While the HDI has the great merit of representing a multidimensional index opposite to the traditional unidimensional evaluation and of being able to give a general idea of the wealth of a population, it does not fully estimate the status of health of that population and does not investigate the socioeconomic health determinants, in other words, it does not inform the politicians and policy-makers on how the status of development of the population (mostly evaluated in the economic dominions such as gross income) affects the health of that population. Hence the need to develop a specific index for health.

Like “development”, also the concept of “health” is a multidimensional abstraction that can hardly be defined by a single component. The World Health Organisation has moved from the definition of health as “absence of disease” to that of “physical, mental and social well-being” [12], thus introducing the key concept that the social environment is one of the basic components of health. Furthermore, the social dominion is the one that is at the basis of the two other components of “health”, namely the physical and mental well-being. It has been finally accepted that society and social environment affect the reality and perception of health and that acting on social and economic factors may yield an improvement of the status of health. It is therefore crucial to have a complete knowledge of the socioeconomic factors, or “indicators”, affecting the status of health of a population, better if those indicators can be grouped under a single “index”, able to give a numeric immediate information, easily understandable and comparable. The ‘composite index of health’ (CIH) we hereby presented combines statistical rigor with high level of communicability and is useful to facilitate comparison and analysis of the status of health of a population at local and national level. However, in-depth studies should be based on the analysis of single indicators.

The CIH has been internally validated on the Italian population and the evident North-South CIH gradient is a further demonstration of the well-known social, cultural and financial disparities within the Country. Despite being quite autonomous social and political entities, neighbouring provinces often share the same cultural background in particular in the South. It is therefore possible to identify spatial patterns able to describe areas of “multidirectional dependence”, where contiguous areas show similar levels of the same phenomenon.

Improving the general status of health of the population would entail tackling the disparities by means of “upstream” changes directed to change the social-environmental determinants of health rather than health itself [3], although an improvement of local healthcare system would also act as a driver to increase the social wellbeing and would back up the improvement of the other indicators.

The governance framework to the global determinants of health should have a “systemic” and conjoined approach involving all the stakeholders and all the national and local policies [3]. Improving the determinants of health would be of great advantage not only to the individuals but also, we may say “mostly”, to the society in terms of improved productivity, increased tax revenue and lower welfare and healthcare costs [12]. The UK Marmot Report identified six objectives of the political choices: (a) improving the health of children, (b) maximizing each person capabilities, at every age, (c) creating good work environments, (d) ensuring healthy lifestyles, (e) improving the status of the environment, (f) creating policies and procedure for medical prevention [13].

Clearly, reaching these objectives would require a thorough knowledge of the determinants of health within the society and within each layer of it.

Although primarily conceived to aggregate the determinants of health of a population and not to directly evaluate their impact on the health of a population, the CIH would be useful to estimate the general status of health as determined by social and financial factors and to guide government actions on non-health issues which may yield positive health outcomes.

Limitation of this study is the lack of an external validation of our CIH. Further studies may be necessary to confirm the applicability of the CIH to other Countries.

## Conclusion

The CIH is a good aggregate of the determinants of health of a population. It is an indirect indicator of the impact of those determinants on the status of health of a population at local and national level and may be extremely useful to explore the relations between health and socioeconomic factors and also to evaluate the influence of proximity with high or low-performing regions on the health of citizens of a specific region or province. Although hereby validated on the Italian population, the CIH should be easily generalised to any other Country.

## Data Availability

Data and material used for this study are publicly available on the Italian Istitute of Statistics website (www.istat.it) and are available from the corresponding Author upon reasonable request.

## Abbreviations

CIH: Composite Index of Health
COMIC: COMposite Indices Creator
ESWB: Equitable and Sustainable Well-Being
LISA: Local Indicator of Spatial Association
ISTAT: National Italian Institute of Statistics
AMPI: Adjusted Mazziotta-Pareto Index
HDI: Human Development Index
GDP: Gross Domestic Product
